# Development and Challenges of Biphasic Membrane‐Less Redox Batteries

**DOI:** 10.1002/advs.202105468

**Published:** 2022-04-04

**Authors:** Xinyu Li, Zhenbo Qin, Yida Deng, Zhong Wu, Wenbin Hu

**Affiliations:** ^1^ Key Laboratory of Advanced Ceramics and Machining Technology of Ministry of Education Tianjin University Tianjin 300072 China; ^2^ Key Laboratory of Composite and Functional Materials School of Material Science and Engineering Tianjin University Tianjin 300072 China; ^3^ Joint School of National University of Singapore and Tianjin University International Campus of Tianjin University Binhai New City Fuzhou 350207 China

**Keywords:** biphasic, energy storage, membrane‐less redox batteries

## Abstract

Ion exchange membranes (IEMs) play important roles in energy generation and storage field, such as fuel cell, flow battery, however, a major barrier in the way of large‐scale application is the high cost of membranes (e.g., Nafion membranes price generally exceeds USD$ 200 m^−2^). The membrane‐less technology is one of the promising approaches to solve the problem and thus has attracted much attention and been explored in a variety of research paths. This review introduces one of the representative membrane‐less battery types, Biphasic membrane‐less redox batteries that eliminate the IEMs according to the principle of solvent immiscibility and realizes the phase splitting in a thermodynamically stable state. It is systematically classified and summarizes their performances as well as the problems they are suffering from, and then several effective solutions are proposed based on the modification of electrodes and electrolytes. Finally, special attention is given to the challenges and prospects of Biphasic membrane‐less redox batteries, which could contribute to the development of membrane‐less batteries.

## Graphical abstract

Ion exchange membranes play important roles in energy generation and storage field. However, a major barrier in the way of large‐scale application is the high cost of membranes. The Biphasic membrane‐less redox battery is a new strategy recently proposed and it is the most likely to achieve large‐scale applications among many membrane‐less strategies.

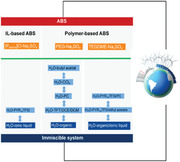

## 1 Introduction

**Figure 1:**
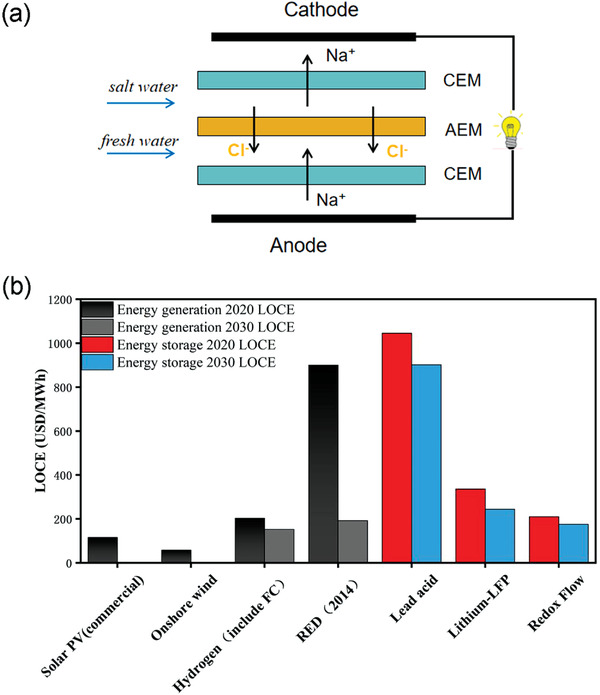
a) RED principle. CEM (Cation‐exchange membrane), AEM (Anion‐exchange membrane). b) LOCE index of some energy generation and storage technologies in 2020 and forecast in 2030 (Energy generation power is more than 1 MW, discount rate is 10%, Energy storage power is based on 100 MW, 10 h system). Data acquired from ref. [2, 3, 13].

Due to the increasingly serious problems in environmental pollution and global warming caused by the large‐scale use of fossil fuels, it is urgent to develop renewable clean energy and sustainable energy conversion and generation technologies. Reverse electrodialysis (RED),^[^ ^1^ ^]^ Redox flow battery (RFB), Fuel cell (FC) are three common energy conversion technologies based on IEMs. In RED (as schematically depicted in **Figure** **1**a), IEMs are placed between salt solution and fresh water to conduct on the conversion of chemical potential and electrical energy by means of concentration difference, achieving the selectively pass of Na^+^ and Cl^‐^. In contrast to RED technology, protons pass freely through IEMs among FC and RFB while the positive and negative reactants were separated. As an important component of these batteries, however, the high cost of IEMs has seriously hindered the broad deployment of these technologies. Alexandros Daniilidis et al.^[^ ^2^ ^]^ has assessed the impact of various factors in RED on Levelized Cost of Energy (LCOE, a measurement used to calculate the average total cost of building and operating the asset per unit of total electricity generated over an assumed lifetime that compares the combination of capital costs, operations and maintenance, performance, and fuel costs) as shown in Figure 1b, and pointed out that the current LOCE of shown in Figure 1b, and pointed out that the current LOCE of RED is USD$ 900 MWh^−1^ due to high cost of membrane and low power density, which far exceeds the cost of other new energy such as solar energy (USD$ 116 MWh^−1^) and wind energy (USD$ 58 MWh^−1^).^[^ ^3^ ^]^ The cost of membrane should be reduced by more than 80% to meet commercial demand, which means that the LCOE should reduce to almost USD$ 192 MWh^−1^, close to the LOCE of hydrogen energy.

**Figure 2:**
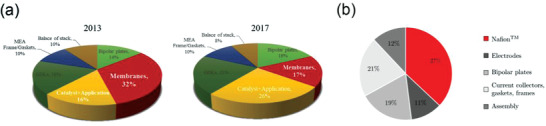
a) Breakdown of the 2013 and 2017 projected FC stack cost at 1000 per year production of 80 kW systems. Data aquired from ref. [5, 6] b) cost structure of 250 kW of VRFB stack with Nafion. Reproduced with permission.^[^ ^8^ ^]^ Copyright 2017, Elsevier.

FC have emerged as a promising candidate for clean energy due to its low level LOCE index,^[^ ^3^ ^]^ and both the catalyst and membranes are the main sources of cost, of which membranes account for 20%≈30%.^[^ ^4^ ^]^ Owing to continuously research on IEMs,^[^ ^5^ ^]^ the cost of membranes has dropped to 17% during 2013 to 2017 as shown in **Figure** **2**a,^[^ ^6^, ^7^ ^]^ but it still occupies a large proportion. In RFB, IEMs (mainly Nafion membrane) cost based on vanadium redox flow battery (VRFB) has reached 20≈40% of battery cost (Figure 2b).^[^ ^8^, ^9^ ^]^ At present, the price of Nafion 117 membrane ranges from USD$ 500≈1000 m^−2^ according to the analysis by Minke et al.^[^ ^10^, ^11^ ^]^ Whereas ideal membrane cost is USD$ 50 m^−2^ or less,^[^ ^12^ ^]^ it is far from the actual value, and so its cost needs to be further reduced to achieve the LOCE expectations in 2030.^[^ ^13^ ^]^

For this purpose, membrane‐less strategy including of “Catalyst selectivity”, “Hybrid flow”, “Laminar Flow” and “Biphasic system” has been proposed to cut costs and realize the large‐scale application of the aforementioned energy conversion technology.

**Figure 3:**
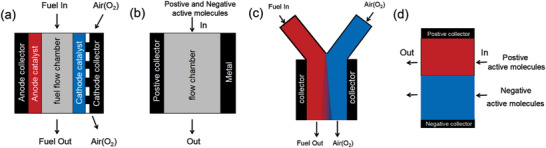
The schematic diagram of a) “Catalyst selectivity” fuel cell. b) Hybrid flow battery. c) Laminar Flow battery. d) Biphasic system battery.

In “Catalyst selectivity” and “hybrid flow” strategies, electrolytes are mixed (depicted in **Figure** **3**a and Figure 3b), while “Laminar Flow” and “Biphasic system” is stratified. Figure 3c and Figure 3d show the electrolyte close the positive and negative electrode is separated from each other by the liquid‐liquid interface. In “Catalyst selectivity” strategy (depicted in Figure 3a), air (or oxygen) enters from the channel near the cathode collector, while fuel flows in through the channel between the cathode and anode catalyst. Oxygen reduction reaction (ORR) should be conducted under cathode catalyst, which has no catalytic activity (or react extremely slow ^[^ ^14^, ^15^ ^]^) for the anode reaction with fuel, and thus realizing the reaction selectivity without membrane. This type of battery is also called direct liquid fuel cells, and some small molecular organics can be employed as fuels coupled with proper catalyst, such as methanol, ethanol, ethylene glycol, glycerol, and other small molecule or their salts.^[^ ^16^, ^17^, ^18^ ^]^ In addition, the principle of catalyst selectivity is also applicable to microbial fuel cells, such as enzyme‐selective catalysis, which is applied to glucose/oxygen biofuel cells,^[^ ^19^ ^]^ ethanol/oxygen biofuel cells^[^ ^20^ ^]^ etc. Although the strategy has eliminated IEMs, it was strongly dependent on the reliable anode catalyst based on expensive and rare noble metals. Low‐ cost and non‐noble‐metal anode catalysts should be explored for the specific fuels in the future.

Figure 3b shows the schematic of membrane‐less hybrid flow battery (MHFB), also called “single flow battery”, where metal used as the negative electrode and mixed electrolyte as a whole channel. It was first proposed by Pletcher,^[^ ^21^ ^]^ and Pb^2+^ in aqueous methanesulfonic acid was applied to deposit PbO_2_ and Pb on the positive and negative electrode during charge process, respectively, then dissolute to Pb^2+^ when discharged. Hence, no membrane was required, thus reducing the cost significantly. Till now, positive and negative reactants of MHFB were limited with Pb‐PbO_2_, Cu‐PbO_2_,^[^ ^22^ ^]^ Zn‐Ni(OH)_2_,^[^ ^23^ ^]^ Zn‐Ce,^[^ ^24^ ^]^ Zn‐Br_2_,^[^ ^25^ ^]^ Zn‐MnO_2_,^[^ ^26^ ^]^ lithium‐polysulfide,^[^ ^27^ ^]^ Metal dendrite growth^[^ ^23^ ^]^ and the resulting uneven distribution of the deposition layer reduced the cycle stability, which limited energy density of MHFB, so their large‐scale applications were also severely restricted. Another membrane‐less method is based on aqueous solution separation by means of laminar flow principle^[^ ^28^ ^]^ as shown in Figure 3c. The laminar flow at low Reynolds number allows liquid streams containing different concentrations of substances to flow side by side through the microchannel, and the ions exchange occurs at the liquid‐liquid interface through diffusion driven by concentration gradient.^[^ ^29^, ^30^ ^]^ A series of such batteries has been developed including of Single‐phase co‐Laminar Flow (SLF) battery,^[^ ^31^, ^32^, ^33^, ^34^ ^]^ Flowing electrolytes separated by a flowing stream of a Supporting Electrolyte (FSE) multi‐stream laminar battery,^[^ ^35^, ^36^ ^]^ Multiphase co‐Laminar Flow (MLF) battery^[^ ^37^, ^38^ ^]^ etc. The power density of laminar flow batteries has been greatly improved up to 700 mW cm^−2^. Laminar flow, however, only occurs in small devices (10^‐1^≈10^2^ cm^2^) with low flow rate (≈1 uL min^−1^),^[^ ^39^, ^40^ ^]^ small characteristic length of the object (≈20 mm),^[^ ^39^ ^]^ and large viscosity, as a result of the application of such batteries limited to micro fuel cells^[^ ^41^ ^]^ and microbial fuel cells.^[^ ^42^ ^]^

In this review, a new type of membrane‐less technology, “Biphasic system” strategy is the emphasis (Figure 3d). It is based on the principle of immiscibility to form a stable liquid‐liquid interface, separating different active molecules. Unlike laminar flow, “Biphasic system” battery can be designed to large size equipment due to static and stable phase splitting for large‐scale energy storage, and there is no requirement for catalyst selectivity, as long as the active molecules that can be separated to form a battery. In addition, the introduction of a non‐aqueous phase with different properties from water can to some extent avoid many problems encountered in aqueous batteries, such as Hydrogen evolution reaction (HER), Oxygen evolution reaction (OER), and broaden the choice of electrode materials (active molecules). The article describes the principle of phase splitting, and lists the types and performance of various Biphasic membrane‐less redox batteries. Due to differences in active molecules, solvent compositions and supporting salts, the batteries exhibit noticeable differences in coulomb efficiency, cycle performance and power density. Besides, problems, challenges (such as cross‐contamination, low power performance and so on) and the corresponding countermeasures are also summarized, which could provide new ideas for the development of membrane‐less batteries technology.

## 2 Types and Principle of Liquid‐Liquid Membrane‐Less Batteries

**Figure 4:**
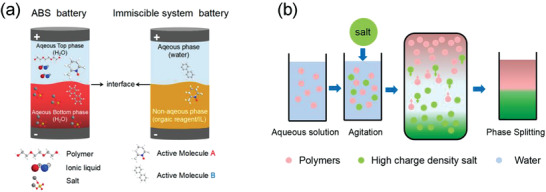
a) ABS battery and immiscible system battery. b) ABS phase splitting principle.

According to the principle of phase splitting, liquid‐liquid membrane‐less batteries could be divided into two types: Aqueous biphasic systems (ABS) battery and Immiscible system battery (**Figure** **4**a).

When the polymer (or ionic liquid, IL) and salt are added to water, the solution will be stratified to form phase‐splitting through the salting‐out effect,^[^ ^43^, ^44^, ^45^, ^46^, ^47^ ^]^ the system is called ABS. Specifically, as shown in Figure 4b, part of the water molecules would combine with the water‐soluble polymer to hydrate with salt ions due to the preferential hydration of the high charge density salt over the water‐soluble polymer when high charge density salts are added to aqueous solutions of “Water‐soluble” polymer. As a result, the solution is separated into polymer‐rich phase and salt‐rich phase. When the polymer is replaced with the IL, the principle is also similar. Then, two groups of active molecules with significant different solubility in the two phases are added to the stratified ABS to finally form the battery electrolyte. At present, the ABS components that can form batteries are consisting of a polymer and a salt as well as an IL and a salt.^[^ ^48^, ^49^, ^50^ ^]^

According to the Like Dissolves Like Rule,^[^ ^51^ ^]^ immiscible system is mainly composed of polar (mainly water) and non‐polar solvents (mainly organics, such as carbon tetrachloride (CCl_4_), nitrobenzene (NB), dichloroethane (DCE), propylene carbonate (PC) and so on). In addition, non‐polar solvents also can be hydrophobic ILs, and its hydrophobic principle is related to the polarity of anions and cations. The immiscible system battery generally has the characteristic that one redox active molecule is confined in the water phase while the other in the ionic liquid or organic phase. Due to the large difference in solubility, the two phases would spontaneously separate with each other to form a thermodynamically stable system in the absence of IEMs (Figures 4a).

### 2.1 Aqueous Biphasic Systems

ABS batteries could be built by two steps and the initial one is to find a proper polymer or IL to form phase‐splitting against with the corresponding salt phase. And then it is should be sought for suitable active molecules that have solubility differences in the above solute to build ABS battery.

#### 2.1.1 ABS Formation Ability

**Figure 5:**
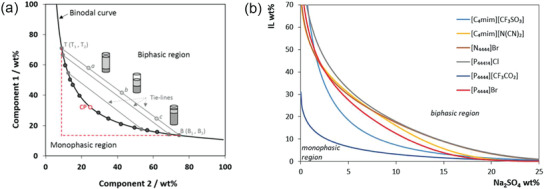
a) Scheme of an orthogonal ternary phase diagram composed of component 1, component 2, and water (in weight fraction, wt%) and the respective binodal curve, TB is tie‐line, moving along the same tie‐line (TL), the concentration of the phases remains the same, differing only in the total compositions and phase volume ratios. Reproduced with permission.^[^ ^54^ ^]^ Copyright 2020, Elsevier. b) Phase diagrams in weight percentage and Chemical structure of six kinds of ILs chosen to form ABS. Reproduced with permission.^[^ ^48^ ^]^ Copyright 2020, Wiley‐VCH.

Suitable solute for ABS should be determined by the solubility curve in the phase diagram (**Figure** **5**a), which is measured by cloud‐point titration.^[^ ^52^, ^53^ ^]^ The curve (binodal) separates the biphasic region (above the curve) from the monophasic region (below the curve).^[^ ^54^ ^]^ In the ABS composed of ionic liquid and salt, the larger the biphasic region is (the closer to the axis origin a binodal curve is), the stronger the ABS formation ability is (phase splitting ability),^[^ ^43^ ^]^ which not only affects the composition of the solution during layering, but also the phase splitting ability. It is related to the hydrophobicity of the ionic liquid (salt‐in ability) and the resulting dissolution of active molecules. Therefore, the ionic liquid with stronger phase splitting ability should be selected. Paula Navalpotros^[^ ^48^ ^]^ has tested the possibility of forming two‐phase systems by coupling six kinds of ILs with Na_2_SO_4_, and the corresponding ability to form ABS as shown in Figure 5b follows the order: [P_4444_][CF_3_CO_2_] > [C_4mim_][CF_3_SO_3_] > [P_4444_]Br > [C_4mim_][N(CN)_2_] > [P_44414_]Cl ≈ [N_4444_]Br. This trend shows the relationship between the hydrophobicity of ionic liquids and the phase splitting ability, and hence high‐hydrophobic ionic liquids are preferred. Of course, the cost should also be considered in the final choice.

#### 2.1.2 Active Molecules Selection in ABS Battery

**Figure 6:**
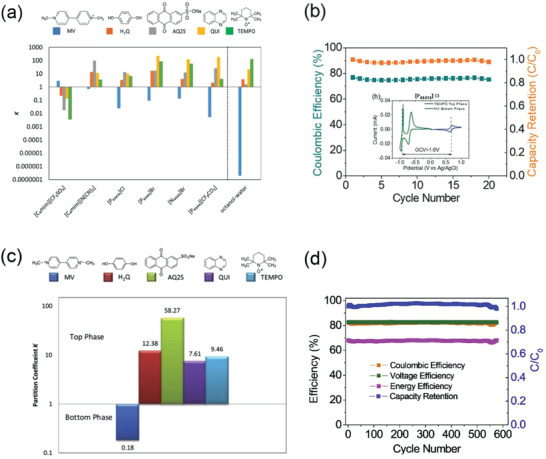
a) Partition coefficients of the target molecules in ABS based on different ionic liquids; b) CV of each phase for the system based on P_44414_Cl + Na_2_SO_4_ with different MV+TEMPO at 20 × 10^‐3^ m concentration and Cycling stability test at 0.16 mA cm^−2^. Reproduced with permission.^[^ ^48^ ^]^ Copyright 2020, Wiley‐VCH. c) Partition coefficients of redox organic molecules between the PEG and salt‐rich phases in the studied ABS; d) Cyclability test of the battery (5% SOC), charging at C/2 and discharging at C. Reproduced with permission.^[^ ^49^ ^]^ Copyright 2020, Elsevier.

According to the selected solute, active molecules should be determined by the solubility differences in the two phases. The constant that measures this difference in solubility is called the partition coefficient K (K=[target molecule 1]_top phase_/[target molecule 2]_bottom phase_). The partition coefficient of one active molecule is more than 1 and the other is less than 1. And the greater the difference between the two values is, the better the selective separation of the two active molecules becomes. Paula Navalpotro tested various ABS batteries consisting of organic molecules coupling with ILs of different partition coefficients (**Figure** **6**a).^[^ ^48^ ^]^

Electrochemical tests such as potential and reversibility of different active molecules were also carried out, and various factors including of solubility and price were integrated, it was chosen of the IL ([P_44414_]Cl) and Na_2_SO_4_ to form a two‐phase system, MV (1,1“‐Dimethyl‐4,4”‐Bipyridinium dichloride) and TEMPO (2,2,6,6‐Tetramethylpiperidine‐1‐oxyl) as active molecules. And the theoretical potential difference was up to reach 1.6 V, with the Coulombic Efficiency (CE) of 80% within 25 cycles and the capacity retention rate of 90% (Figure 6b).

Further, in order to reduce costs, Paula Navalpotro also used polyethylene glycol (PEG, a kind of polymer) instead of ionic liquid to form ABS,^[^ ^49^ ^]^ and its corresponding partitions coefficients coupling with several active molecules were shown in Figure 6c. It could be found that only MV preferentially partitioned to the bottom salt‐rich phase of the ABS (K≪1), where MV and TEMPO used as positive and negative active molecules, respectively, considering the battery voltage (1.23 V) and the excellent reversibility of TEMPO. This ABS exhibited the capacity retention rate up to 99.99% when cycling at 5% capacity utilization for 550 times. Compared with ILs, PEG has the characteristics of low cost, environmentally friendliness and large‐scale production, which has great advantages in large‐scale energy storage.

**Figure 7:**
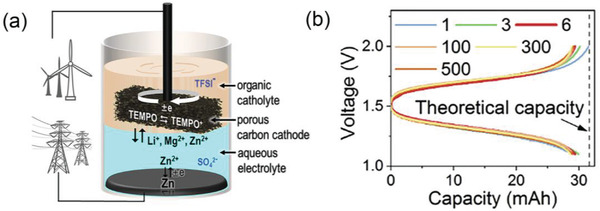
a) Schematic illustration of the Stirred Self‐Stratified Battery (SSB); b) Cycling performance of the SSB at a stirring speed of 80 rpm at a lower concentration (44mM). Reproduced with permission.^[^ ^50^ ^]^ Copyright 2020, Cell Press.

In addition, the strategy of electrode stirring was introduced (**Figure** **7**a) by Yunhui Huang^[^ ^50^ ^]^ to improve the battery performance with the capacity utilization rate more than 90% at a lower concentration of 44 mM (TEMPO) as shown in Figure 7b. In this battery, another polymer, tetraethylene glycol dimethyl ether (TEGDME) was applied coupled with MgSO_4_ to construct an ABS, where TEMPO used as the organic phase active molecule and Zn/ZnSO_4_ as the aqueous electrolyte. It also exhibited the best power performance of membrane‐less battery (about 40 mW cm^−2^) so far with stable charging and discharging process as well as high CE of 99% when cycling for two months at a current of 1 A.

### 2.2 Immiscible System

Different from the ABS battery consisting of aqueous phase, the immiscible system is composed of aqueous phase and non‐aqueous phase, and it also could be divided into water‐ionic liquid‐based battery and water‐organic solvent‐based battery according to the characteristic of the non‐aqueous phase.

#### 2.2.1 Water‐Ionic Liquid‐Based Battery

**Figure 8:**
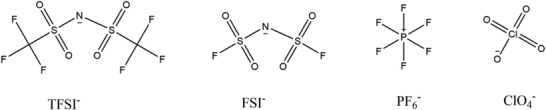
Structures of TFSI^‐^, FSI^‐^, PF_6_^‐^, ClO_4_^‐^.

Hydrophobic ILs could be separated from water due to its non‐polar characteristics attributed from non‐polar anion (such as TFSI^‐^, FSI^‐^, PF_6_^‐^, ClO_4_^‐^ and so on, as shown in **Figure** **8**) coupling with hydrophobic cations of carbon chain. And the longer the length of carbon chain, the stronger the hydrophobicity is. Due to the non‐polar characteristics of hydrophobic ILs, it will be separated from water. This type ILs are more common in the combination of long‐chain cations and anions containing TFSI^‐^, FSI^‐^, PF_6_^‐^, ClO_4_^‐^ and so on. This is because the structure of anions are non‐polar (as shown in Figure 8) compared with water. In addition, the carbon chain is hydrophobic chain, the longer the length, the stronger the hydrophobicity. Therefore, both the non‐polar anion and long‐chain cations resulted in the immiscibility between hydrophobic IL and water, which is a necessary prerequisite to build Water‐ionic liquid‐based battery.

**Figure 9:**
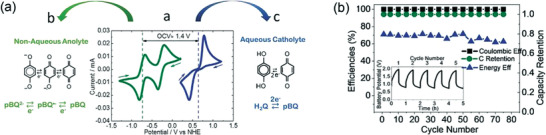
a) Open circuit potential of the battery and the conversion of active molecules in each phase. b) Cyclability study at ±0.2 mA cm^−2^. Coulombic efficiency, energy efficiency, and capacity retention versus cycles. Inset: Voltage profile of the battery for the first 5 cycles. Reproduced with permission.^[^ ^55^ ^]^ Copyright 2017, Wiley‐VCH.

An immiscible system battery was first developed by Rebeca Marcilla^[^ ^55^ ^]^ applied with a the hydrophobic IL, 1‐butyl‐1‐methylpyrrolidinium bis(trifluoromethanesulfonyl)imide (Pyr14TFSI). Benzoquinone (BQ) was used as the active molecule, which was hydroquinone (H_2_Q) in acidic aqueous solution and parabenzoquinone (pBQ) in Pyr14TFSI. The open circuit potential of the battery was 1.4 V. Since the active molecule can be converted with each other in the two phases (**Figure** **9**a), there was no cross‐contamination problem, and the CE of the battery could be maintained at 100% within 80 cycles (Figure 9b). Because of the low conductivity (2.2 mS cm^−1^) and high viscosity of the ionic liquid (84.33 cP), however, the current density of the battery was relatively lower (0.025≈0.4 mA cm^−2^), leading to lower power performance. Besides, the price of the most of ionic liquid, especially for Pyr14TFSI is usually expensive, resulting the high cost of the battery.

Since ILs are designer solvents, they can be based on ions of a very wide range regarding costs. So, ionic liquids normally fall in the range of 5≈20 times more expensive than organic solvents, on a laboratory scale, but there are those which are cheaper, and those which are much more expensive.^[^ ^56^, ^57^ ^]^ However, ILs that require specific anions for hydrophobicity are generally more expensive, especially when coupled with the bis{(trifluoromethyl)sulfonyl} amide (or bistriflamide) ion.^[^ ^56^ ^]^

Although hydrophobicity ILs are expensive, water‐ionic liquid‐based battery remain a valuable research direction. At present, the synthesis and production process of ionic liquids is continuously improved, and the price is continuously reduced;^[^ ^58^ ^]^ As it is at the heart of green chemistry to recycle ionic liquids, 10≈20 recycles gives them the same cost per cycle as conventional organic solvents, and over 50 recycles makes them significantly cheaper;^[^ ^56^ ^]^ ILs has many other advantages such as nonvolatile, non‐flammability, wide potential window, and which means could potentially be expand many applications in other battery systems such as lithium metal, incombustible metal‐ion battery and other wide‐potential metal‐ion batteries.^[^ ^59^ ^]^

#### 2.2.2 Water‐Organic‐Based Bttery

Due to the high cost of ILs, researches have been tried to replace the IL with cheaper organic solvent. In addition, organic solvent exhibits the characteristics of low viscosity and the resulting enhanced conductivity, thereby improving battery performance. In general, water‐organic‐based batteries could be divided into two categories. One type is composed of only one active molecule, such as BQ mentioned above. The other is consisting of two different active molecules dissolved in different phases, respectively, and metallic zinc is also used as the negative electrode instead of carbon collector for the second type in some situation.

**Figure 10:**
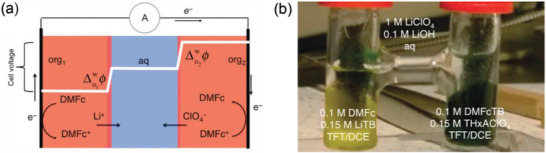
a) Battery reactions upon discharge and the illustration of the Galvani potential profile in the cell. b) figure showing the realization of the battery and the composition of the electrolytes in the fully charged state. Reproduced with permission.^[^ ^60^ ^]^ Copyright 2016, The Royal Society of Chemistry.

The first type water‐organic‐based battery was studied in 2016. Hubert H. Girault has developed the water‐organic‐based battery with only one.^[^ ^60^ ^]^ Active molecule, decamethylferrocene (DMFc), and then its performance was evaluated in the two types of solvents, trifluorotoluene (TFT) and 1,2‐dichloroethane (DCE). The battery structure is shown in **Figure** **10**a. Both sides of the battery are the same organic solvent, and the middle is the water layer. LiClO_4_ as supporting electrolytes was added to the water phase, playing the roles of constituting a pathway, exchanging ions to maintain charge balance and improving conductivity with the exchange ions of Li^+^ and ClO_4_^‐^. The aqueous solution with dissolved LiClO_4_ salt and the organic solution with dissolved lithium tetrakis pentafluorephenylborate (LiTB) salt on the left will form a Galvani potential $\Delta_{O1}^{W}\Phi$ at the interface due to difference in energy of the carriers in both phases (chemical potentials), and the same on the right will form Galvani potential $\Delta_{O2}^{W}\Phi$ between LiClO_4_ aqueous solution and tetrahexylammonium perchlorate (THxAClO_4_) organic solution. The total battery potential is the sum of the active molecules potential differences (ΔE) and the Galvani potential($\Delta_{O1}^{W}\Phi$+$\Delta_{O2}^{W}\Phi$). The battery charging and discharging cycle experiment was carried out on two porous glass carbon electrodes (radius 1.5 mm), The whole electrolytic cell was H‐shaped as shown in Figure 10b, and the EE of using DCE and TFT solvents are 83% and 65%, respectively, at current 0.3 mA (about 4.2 mA cm^−2^). This battery design exhibited good isolation of the active molecule, achieving the similar effect of ion exchange membranes. The CE of 20 cycles was close to 100% and the Energy Efficiency (EE) close to 80% in DCE. However, the cycle stability was limited by the evaporation of organic solvents.

**Figure 11:**
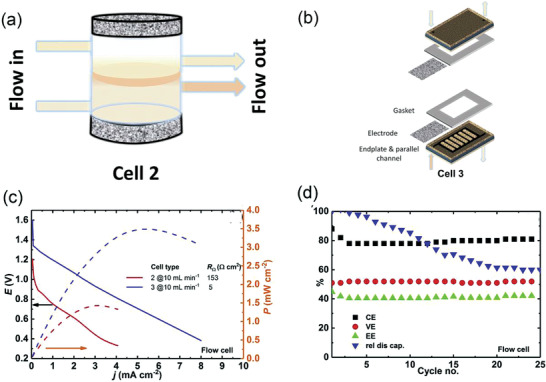
a) Flowing‐electrolyte cylindrical cell with graphite foil electrodes; b) conventional planar flow cell (Fuel Cell Technologies) with carbon paper electrodes; c) polarization curves obtained with Cell 2 (static) and Cell 3 (flow cell). d) CE, VE (voltage efficiency), EE and relative discharge capacity over 25 cycles for Cell 3 cycling with constant‐current charging‐discharging at 1 and 0.2 mA cm^−2^, respectively. Reproduced with permission.^[^ ^61^ ^]^ Copyright 2018, Elsevier.

In 2018, Saif Almheiri^[^ ^61^ ^]^ developed an all‐iron membrane‐less battery including of two active molecules, FeSO_4_ and Fe(acac)_3_ dissolved in water and ethyl acetate with IL, Pyr14TFSI (**Figure** **11**a). K_2_SO_4_ was used as a supporting salt to balance the charge. Furthermore, fluidity method was first introduced in biphasic system battery (Figure 11b). With a flow rate of 10 mL min^−1^, the current density could reach 3 mA cm^−2^ (Figure 11c). Owing to the unstable characteristics of the active molecules, unfortunately, the battery decayed too fast, up to more than 30% within 25 cycles (Figure 11d).

**Figure 12:**
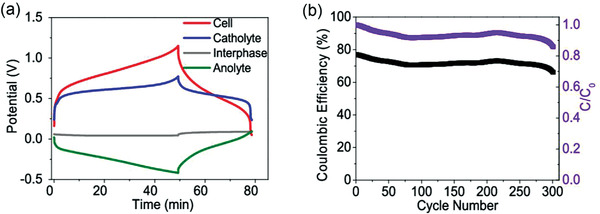
Active species concentration: 0.1 m in catholyte and anolyte a) Galvanostatic charge‐discharge cycle at 1 mA cm^2^ for charging and 2 mA cm^−2^ for discharging (SOC 15%); b) Cycling stability (5% SOC) at 1 mA cm^−2^ for charging and 2 mA cm^−2^ for discharging. Reproduced with permission.^[^ ^62^ ^]^ Copyright 2018, ACS.

In order to seek for suitable batteries and exploring the versatility of biphasic battery, Rebeca Marcilla conducted a lot of screening on active molecules and solvents.^[^ ^62^ ^]^ Active molecules such as 2,3‐dimethylanthraquinone (2,3‐DMAQ), 1,4‐bis(pentylamino) anthraquinone (OilBlue N), 4‐hydroxy‐2,2,6,6‐tetramethylpiperidine1‐oxyl (OH‐TEMPO), 2,2,6,6‐tetramethylpiperidine‐1‐oxyl (TEMPO) were tested to construct a serious of biphasic systems including of water‐butanone, water‐PC, water‐(PC+Pyr14TFSI). Among them, 0.1 m OH‐TEMPO/pBQ dissolved in water‐(PC+Pyr14TFSI) system showed a better cycle performance. The capacity retention rate was above 80% when 300 cycles at 2 mA cm^−2^ under 5% SOC (**Figure** **12**b).

**Figure 13:**
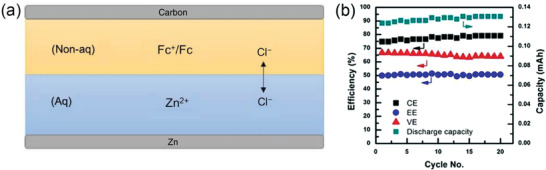
a) Design of the membrane‐less zinc‐ferrocene battery. b) CE), VE, EE, and discharge capacity for each cycle in the 20 cycles test. Reproduced with permission.^[^ ^63^ ^]^ Copyright 2017, ECS.

In addition, the effect of negative electrode was also studied, and metallic zinc was used as a negative electrode instead of carbon collector by Ke Gong.^[^ ^63^ ^]^ Besides, ZnCl_2_ aqueous solution was used as the negative electrolyte, to form water‐organic‐based battery with ferrocene (Fc) dissolved in butyl acetate as positive electrolyte And IL salt (Aliquat 336) as the supporting electrolyte (Figure 13a). Due to the excessive polarization, however, the capacity utilization rate was less than 10% even at a relatively low current density (0.1 mA cm^−2^), and the CE was less than 80% within 20 cycles (**Figure** **13**b).

**Figure 14:**
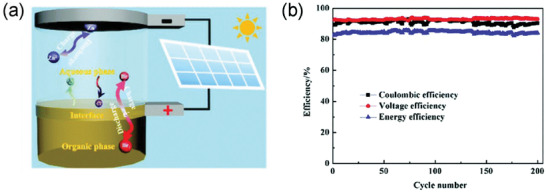
a) The schematic diagram of the interfacial battery. b) The charge‐discharge cycling performance of the interfacial battery at 5 mA cm^−2^. Reproduced with permission.^[^ ^64^ ^]^ Copyright 2018, The Royal Society of Chemistry.

Huamin Zhang^[^ ^64^ ^]^ also used zinc as the negative electrode, while in the choice of positive active molecules, the non‐polar molecule, Br_2_ was chosen as the positive active molecule to dissolve in non‐polar solvent CCl_4_. According to The Like Dissolves Like Rule, Br_2_ was almost completely dissolved in CCl_4_, but not dissolved in water, and thus self‐discharge inducing by the reaction between Br_2_ and Zn could be avoided. **Figure** **14**a showed the structure and reaction of the battery. During the charging process, the reaction of the positive and negative electrodes is as follows.

(1) $${\mathrm{Zn}}^{2+}+2e^{-}↔\mathrm{Zn}\;E=-0.76\;V\;\left(\mathrm{vs}\;\mathrm{SHE}\right)$$

(2) $$2{\mathrm{Br}}^{-}↔{\mathrm{Br}}_{2}+2e\;E=1.076\;V\;\left(\mathrm{vs}\;\mathrm{SHE}\right)$$

3 m KCl was used as the supporting electrolyte, and the battery has a CE of 96% and an EE of 81%, respectively. Besides, it also exhibited an excellent stability within 200 cycles at a current density of 5 mA cm^−2^ (Figure14b).

**Figure 15:**
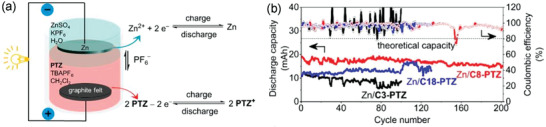
a) Schematic of a stratified, membrane‐less battery; b) Battery performances of stratified Zn/PTZ batteries with 0.5 m C8‐PTZ. Reproduced with permission.^[^ ^65^ ^]^ Copyright 2021, ACS.

In 2021, dichloromethane (DCM, CH_2_Cl_2_) and water were used as immiscible solvents by Jianbing Jiang,^[^ ^65^ ^]^ while Zn/ZnSO_4_ as the negative active molecule to dissolve in water phase and Phenothiazine (PTZ) and its derivatives as the positive one in DCM to form a battery (**Figure** **15**a). In addition, PF_6_^‐^ served as an ion exchange between the two phases. The battery exhibited a CE of 96% with a capacity retention rate of 79.1% after 202 cycles at 20mA (1.5 C) as shown in Figure 15b. It should be noticed that the capacity utilization rate reached 72% of theoretical value (9.72 Ah L^−1^), which was the highest value currently achieved for a biphasic battery under static conditions. Furthermore, the influence of alkyl chains length was conducted on the hydrophobicity of PTZ and its derivatives, the solubility in DCM, as well as the cycling performance. And its hydrophobicity effect was evaluated through solvation free energy and molecular dynamics. Subsequently, the research group also carried out a preliminary test of fluidity relying on the existing system.^[^ ^66^ ^]^

## 3 Challenges of Biphasic Membrane‐Less Redox Batteries

### 3.1 Cross Contamination Limitation

According to **Table** **1**, it can be seen that the biphasic battery currently has a large gap compared with the cycle life of the IEM‐based battery (>1000).^[^ ^67^ ^]^ The main factor that affects the cycle performance is the decaying of the active reactants, induced by cross‐contamination attributing from the similar solubility of active molecules as well as the ion migration. When the difference in the solubility of active molecules is not large enough, it would cause the active molecules to dissolve in the same phase and react with each other in charge and discharge process, as a result of a large loss of capacity. The electrode surface reaction would create active anions or cations during charge and discharge process, and then the migration of these ions between the two phases by the electric field would also cause cross‐contamination and reduce cycle performance. In addition, due to the difference in the concentration of active molecules in the two phases, diffusion across the membrane is inevitable. This crossover will result in the permanent loss of redox active molecules (ions) in one phase, because it cannot return to its original phase, and will eventually lead to capacity loss and capacity ratio distortion of the battery.^[^ ^68^ ^]^ Another result of the crossover is the self‐discharge of the charged redox ions, which is related to the reaction between the diffused active molecules (ions) and the active molecules (ions) of the other phase.^[^ ^69^ ^]^ Based on the summarizes of cross‐contamination, various strategies including of active molecules selection, electrode adsorption, counterion binding and galvanic potential limitation were proposed according to different components of the battery.

**Table 1:** Summarized the types, composition, performance, fluidity of membrane‐less batteries

Type	Battery	Solvent	Exchange Salts	Active Spice	CE	Cycles	Year	fluidity
ABS	MV/TEMPO	H_2_O([P_44414_]Cl)	Na_2_SO_4_	TEMPO/TEMPO^+^	80	25	2018^[^ ^48^ ^]^	×
		H_2_O(Na_2_SO_4_)		MV/MV^2+^				
	MV/TEMPO	H_2_O([31.7% PEG 3.2% Na_2_SO_4_(0.23 m))	Na_2_SO_4_	TEMPO/TEMPO^+^	>82	550	2020^[^ ^49^ ^]^	×
		H_2_O(1.19% PEG 17.75% Na_2_SO_4_(≈1.4 m))		MV/MV^2+^				
	Zn/TEMPO	H_2_O(TEGDME)	MgSO_4_	TEMPO/TEMPO^+^	>99	500	2020^[^ ^50^ ^]^	√
		H_2_O(MgSO_4_)		Zn/Zn^2+^				
Immiscible system	DMFc	TFT/DCE(0.15M LiTB)	LiClO_4_	DMFc/DMFc^+^	99	20	2016^[^ ^60^ ^]^	**√**
		H_2_O(1M LiClO_4_/0.1M LiOH)						
		TFT/DCE(0.15M THxAClO_4_)						
	pBQ/H_2_Q	H_2_O(0.1M HCl)	HCl	H_2_Q/pBQ	99	75	2017^[^ ^55^ ^]^	×
		Pyr_14_TFSI		pBQ^2^/pBQ				
	Zn/Fc	butyl acetate	Aliquat 336	Fc^+^/Fc	75	20	2017^[^ ^63^ ^]^	×
		H_2_O(1M ZnCl_2_)		Zn/Zn^2+^				
	ZnBr_2_	H_2_O(2M ZnBr_2_)	KCl	Br^‐^/Br_2_	96	200	2018^[^ ^64^ ^]^	×
		CCl_4_		Zn/Zn^2+^				
	All‐iron	H_2_O	K_2_SO_4_	Fe^2+^/Fe^3+^	80	25	2018^[^ ^61^ ^]^	**√**
		ethyl acetate(Pyr_14_TFSI)		Fe(acac)_3_/Fe(acac)_3_^‐^				
	(A)2,3‐DMAQ/H_2_Q	H_2_O(0.1M HCl)	HCl	H_2_Q/pBQ	>80	25	2018^[^ ^62^ ^]^	×
		Pyr_14_TFSI		2,3‐DMAQ/2,3‐DMAQ^2‐^				
	(B)OilBlue N/H_2_Q	H_2_O(0.1M HCl)	HCl	H_2_Q/pBQ	≈90	25		
		Pyr_14_TFSI		OilBlue N				
	(C)pBQ/H_2_Q	H_2_O(0.1M HCl)	HCl	H_2_Q/pBQ	/	/		
		2‐Butanone(0.1 m TBAPF_6_)		pBQ^2‐^/pBQ				
	(D)pBQ/H_2_Q	H_2_O(0.1M HCl)	HCl TBAPF_6_	H_2_Q/pBQ	>40	14		
		PC(0.1 m TBAPF_6_)		pBQ^2^/pBQ				
	(E)pBQ/TEMPO	H_2_O(0.1M NaCl )	NaCl	TEMPO/TEMPO^+^	80	25		
		Pyr_14_TFSI		pBQ^2^/pBQ				
	(F)pBQ/OH‐TEMPO	H_2_O(0.5M NaCl )	NaCl	OH‐TEMPO/OH‐TEMPO^+^	<80	300		
		PC(Pyr_14_TFSI)		pBQ^2^/pBQ				
	(G)2,3DMAQ/OH‐TEMPO	H_2_O(0.5M NaCl )	NaCl	/	/	/		
		Pyr_14_TFSI						
	(H)OilBlue N/OH‐TEMPO	H_2_O(0.5M NaCl )	NaCl	/	/	/		
		Pyr_14_TFSI						
	(I)pBQ/OH‐TEMPO	H_2_O(0.5M NaCl )	NaCl TBAPF_6_	/	/	/		
		2‐Butanone(0.1 m TBAPF_6_)						
	(J)pBQ/OH‐TEMPO	H_2_O(0.5M NaCl )	NaCl TBAPF_6_	/	/	/		
		PC(0.1 m TBAPF_6_)						
	Zn/PTZ	H_2_O(0.3 m KPF_6_)	KPF_6_ TBAPF_6_	C8‐PTZ/C8‐PTZ^+^	96%	202	2021^[^ ^65^ ^]^	×
		DCM(0.5 m TBAPF_6_)		Zn/Zn^2+^				
	Zn/PTZ	H_2_O(0.3 m NH_4_PF_6_)	NH_4_PF_6_ TBAPF_6_	C3‐PTZ/C3‐PTZ^+^	99%	194	2022^[^ ^66^ ^]^	**√**
		PC/MeCN(0.5 m TBAPF_6_)		Zn/Zn^2+^				

#### 3.1.1 Active Molecules Selection

Table 1 shows the CE, cycle performance of various Biphasic membrane‐less redox batteries. It can be found that the batteries with better performance (CE more than 95%) can be divided into two categories according to the selection of active molecules selection.

**Figure 16:**
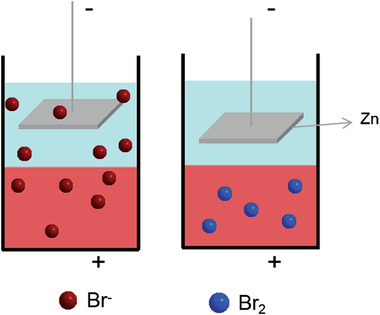
Schematic of distribution of Br_2_ and Br^‐^ in biphasic system.

One is metal‐organic battery, such as Zn/Br_2_,^[^ ^64^ ^]^ Zn/TEMPO,^[^ ^50^ ^]^ with huge difference in the solubility of active molecules in different solvents. The positive electrolyte was generally a strong non‐polar organic solvent (such as CCl_4_) dissolved with a non‐polar active molecule (such as Br_2_), while it was almost insoluble in polar water. Furthermore, the metal electrode was placed far away from the liquid‐liquid interface, and Br_2_ does not move since itself is no charge during the discharge process (**Figure** **16**), greatly reducing the possibility of cross‐contamination. At present, the commonly used metal negative electrode is zinc, which is low cost and high solubility for zinc ion in water. However, it also faces similar problems with other zinc‐based aqueous batteries such as dendrite growth, passivation and hydrogen evolution of zinc.^[^ ^70^ ^]^

The other one is to use a symmetric redox couple, mainly represented by pBQ/H_2_Q.^[^ ^55^ ^]^ As shown in Figure 9a, the same active molecule has different potential in different phase (There is no overlapping part of the potential) and generated ions mutually were converted between the two phases after gaining or losing protons and will not cause permanent capacity loss, just like vanadium batteries, the used electrolyte can simply be restored to its original state after a rebalancing procedure, and the electrolyte can be reused. Symmetric redox flow battery^[^ ^71^ ^]^ has similar principle but only been applied in same phase, however, for another type of battery‐Aqueous organic bipolar redox flow battery,^[^ ^72^ ^]^ the two active molecules are connected by covalent bonds to achieve the above effect, because the choice of two active molecules is more flexible, it will have greater application potential in a Biphasic membrane‐less system.

#### 3.1.2 Electrode Adsorption

**Figure 17:**
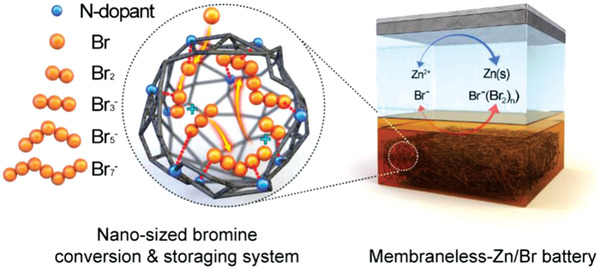
Nanosized Br trapping system for MLFL‐ZBB. Schematics of proposed MLFL‐ZBB employing NGF electrode. Reproduced with permission.^[^ ^73^ ^]^ Copyright 2019, Wiley‐VCH.

The charged active molecules could also be confined to the electrode surface by the electrostatic adsorption, and thus avoiding the cross contamination. For instance, Hee‐Tak Kim^[^ ^73^ ^]^ used protonated pyridinium nitrogen doped microporous carbon to modify the graphite felt, and the resulting abundant positively charged centers in the micropores could effectively capture bromine and polybromine anions, and promoted the conversion of bromine to polybromine through electrochemical growth mechanism (**Figure** **17**), eliminating the bromine crossover. So it showed extraordinary stability within 1000 charge‐discharge cycles with the CE of 90% and the EE of more than 80%. This research provided a new way to solve the cross contamination of active molecules.

#### 3.1.3 Counterion Binding

**Figure 18:**
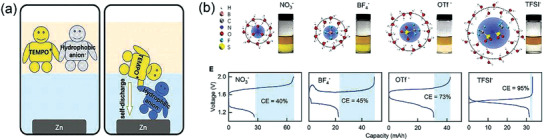
a) Image illustrating the influence of the hydrophobicity of the anion on the TEMPO^+^ distribution. b) Simulated hydration shell structures of different anions, and digital images showing the phase separation effect and Measured first‐round charge‐discharge curves of the SSBs with different anions. The blue bands indicate the capacity loss in the first cycle. Reproduced with permission.^[^ ^50^ ^]^ Copyright 2020, Cell Press.

In 2020, Jintao Meng et al^[^ ^50^ ^]^ proposed a new strategy of counterion selection principle to prevent the positive active ion (TEMPO^+^) from migrating to the negative electrode during discharge, ensuring TEMPO^+^ to confined in the organic phase. A hydrophobic anion, Lithium bis(trifluoromethanesulfonyl)imide (LiTFSI) was applied to combine with TEMPO^+^ (**Figure** **18**a), so as to reduce the solubility of TEMPO^+^ in water. It could be seen that the CE reached more than 95% (Figure 18b) without the cross‐contamination of active ions. LiTFSI, however, is so expensive that its cost occupied the main one of the battery (LiTFSI: USD$ 180 kg^−1^, 97.8% total cost), and thus finding cheap alternatives would be the key task in this strategy.

#### 3.1.4 Galvanic Potential Limitation

For membrane‐less batteries, ion crossover is more likely to occur due to the lack of a physical membrane, many factors such as active molecules diffusion,^[^ ^49^, ^74^ ^]^ ion migration and interface reaction^[^ ^75^ ^]^ would lead to cross contamination, which could be contributed from the Galvanic potential of a certain ion migrate at the liquid‐liquid interface by Hubert H. Girault research.^[^ ^60^ ^]^ The ion transfer mechanism at the interface was investigated that the migration of active ions could be effectively limited by the transfer energy difference of supporting electrolyte ions in two phases.^[^ ^76^, ^77^ ^]^ And the corresponding Galvanic potential was compared with ClO_4_^‐^ (‐0.2v) and Cl^‐^ (0.3v), and then ClO_4_^‐^ was chosen that it could effectively limit the transfer of DMFc^+^ and the CE of battery could reach 100%.

### 3.2 Power Performance Limitation

There are many factors affecting battery power performance, mainly by minimizing the ohmic resistance, maximizing the transport of electrolytes, and boost the surface area and activity of electrodes.^[^ ^78^ ^]^ Traditional water‐based battery electrolyte has high conductivity and low viscosity, and the membrane has good ion conductivity, so it has better power performance, such as commercialized vanadium battery (80≈300 mA cm^−2^),^[^ ^79^ ^]^ Their modification of the battery power is mainly concentrated on the electrode. In contrast, research pays more attention to the electrolyte for organic batteries to improve power density (with low performance).

Compared with above, membrane‐less battery, its current is mainly concentrated in the range of 0.05≈10 mA cm^−2^ as listed in **Table** **2** with relatively lower power performance (0.04≈10 mW cm^−2^), greatly limiting the battery application and increasing the cost. Therefore, the improvement of power performance is very important for the application of membrane‐less battery. At present, there are several factors should be noted including of the conductivity of electrolyte, migration mechanism of interfacial ion and mass transfer process, which are closely related with the power performance of the battery.

**Table 2:** Summarized the power performance of membrane‐less batteries

Battery‐active species	concentration	SOC	Electrode area(cm^2^)	Voltage (Theoretical/actual)	Current density (mA cm^−2^)	Power (mW cm^−2^)
DMFc^[^ ^60^ ^]^	100mM	50	0.07	0.58/0.8	4.2	3.4
pBQ/H_2_Q^[^ ^55^ ^]^	20mM	35	5	1.4/0.8	0.05	0.04
	0.1M	35		1.4/0.9	0.2	0.18
Zn/Fc^[^ ^63^ ^]^	1M/0.1M	1	20	1.16/0.2	0.1	0.02
ZnBr_2_^[^ ^64^ ^]^	2M	/	4	1.85/1.7	15	25.5
	5M	13.6		1.85/1.7	5	8.5
All‐iron^[^ ^61^ ^]^	0.1M	50	5	1.2/0.75	0.2	0.15
MV/TEMPO^[^ ^48^ ^]^	20mM	20	1.5	1.6/1.35	0.16	0.21
(A)2,3DMAQ/H_2_Q^[^ ^62^ ^]^	20mM	35		1.9/1.35	0.16	0.2
(B)OilBlue N/H_2_Q^[^ ^62^ ^]^	20mM	35		2.1/1.7	0.16	0.27
(C)pBQ/H_2_Q^[^ ^62^ ^]^	/	/		/	/	/
(D)pBQ/H_2_Q^[^ ^62^ ^]^	20mM	5		1/0.55	0.16	0.09
(E)pBQ/TEMPO^[^ ^62^ ^]^	40/20mM	5		1.5/1.4	1/2	1.4
(F)pBQ/OH‐TEMPO^[^ ^62^ ^]^	0.1M	5		1/0.6	1/2	0.6
(G)2,3DMAQ/OH‐TEMPO^[^ ^62^ ^]^	20mM	/		2	/	/
(H)OilBlue N/OH‐TEMPO^[^ ^62^ ^]^	20mM	/		2.2	/	/
(I)pBQ/OH‐TEMPO^[^ ^62^ ^]^	20mM	/		1.2	/	/
(J)pBQ/OH‐TEMPO^[^ ^62^ ^]^	20mM	/		1.4	/	/
MV/TEMPO^[^ ^49^ ^]^	0.1M	5	1.5	1.23/0.8	3.2	2.64
Zn/TEMPO^[^ ^50^ ^]^	44mM	>91	1.2	/1.5	25	37.5
	1.5M	37.5	/	1.5/1	/	/
Zn/PTZ^[^ ^65^ ^]^	0.5M	72	3.14	1.67/1.3	6.36	8

#### 3.2.1 Conductivity

Ions conductivity is an important performance index of the battery, determined by the migration ability of ions in the solution. The electrolyte conductivity of the commercial all‐vanadium batteries is basically better in the range of 10^‐1^≈1 S cm^−1^,^[^ ^80^ ^]^ while its membrane ≈10^‐1^ S cm^−1^.^[^ ^81^ ^]^ For membrane‐less battery, supporting electrolyte (usually inorganic salt) is added to improve its conductivity, and this salt has a strong ionization ability in the water phase, which exhibits a relatively high conductivity. While for non‐aqueous solvents, the conductivity is generally 10^‐8^≈10^‐10^ S cm^−1^ due to the high viscosity and incomplete ionization.^[^ ^82^ ^]^ With the addition of supporting electrolyte, the ionic conductivities could be increased up to 10^‐2^ S cm^−1^,^[^ ^83^ ^]^ and **Table** **3** lists the values of conductivity when non‐aqueous solvents dissolved with 1 mol L^−1^ supporting electrolytes.^[^ ^84^ ^]^ Among them, some organic solvents with relatively high conductivity when adding proper supporting electrolyte (3≈6 × 10^‐2^ S cm^−1^), such as acetonitrile, are miscible with water and not suitable for biphasic systems. While the conductivity of water‐immiscible organic solvents is generally low (<2 × 10^‐2^ S cm^−1^), such as DME, DMSO, THF, etc.

**Table 3:** Conductivity of non‐aqueous solvents dissolved with 1 mol L^−1^ supporting electrolytes

Electrolytes	Ionic conductivity ( mS cm^−1^)
1 m TEA‐BF_4_/acetonitrile	56
1.5 m TEA‐BF_4_/acetonitrile	60
1 m TEA‐TFSI/acetonitrile	46
1 m LiBF_4_/acetonitrile	16
1 m LiClO_4_/acetonitrile	34
1 m LiPF_6_/acetonitrile	50
1 m LiTFSI/acetonitrile	36
1 m LiTFSI/diethylene glycol dimethyl ether	8
1 m LiTFSI/1,2‐dimethoxyethane (DME)	14
1 m TEA‐TFSI/1,2‐dimethoxyethane (DME)	17
1 m TEA‐TFSI/dimethyl sulfoxide (DMSO)	9
1 m TEA‐TFSI/tetrahydrofuran (THF)	11
Nafion117^[^ ^85^ ^]^	78

Most of the common inorganic salts cannot be dissolved and ionized in water‐immiscible organic solvents, so some ionic liquids are selected as supporting electrolyte salts to improve the conductivity of the organic phase. According to the data in Table 3, it can be seen that the conductivity of organic phase is generally low, as a result even if ionic liquids or other supporting electrolytes are added to improve conductivity, the current density the corresponding battery is only 0.01≈0.5 mA cm^−2^, leading to low power performance of the organic flow battery.^[^ ^84^ ^]^ In addition, solvent composed entirely of hydrophobic ionic liquids as a non‐aqueous phase is also a way to improve conductivity. The conductivity of ionic liquids acted as solvent but it does still not have the perfect ideal range (10^‐3^≈10^‐2^ S cm^−1^) due to their high viscosity.^[^ ^86^ ^]^ However, Non‐aqueous phase conductivity improved by the above two ways, which is 1 to 2 orders of magnitude different from the water phase in **Table** **4**. So, ion conductivity of the non‐aqeous phase may limit the power performance of the battery.

**Table 4:** Conductivity and power performance of membrane‐less battery

Battery‐active species	Solvent and active species	Conductivity (ms cm^−1^)	Power (mW cm^−2^)
pBQ‐H_2_Q^[^ ^55^ ^]^	0.1M HCl (H_2_Q)	392	0.18
	Pyr_14_TFSI (pBQ)	2.2	
Zn‐Fc^[^ ^63^ ^]^	30 mol.% Aliquat 336/70 mol.% butyl acetate(0.1M Fc)	0.11	0.02
	H_2_O(1M ZnCl_2_)	/	
ZnBr_2_^[^ ^64^ ^]^	H_2_O(2M ZnBr_2_)	250	8.5
	CCl_4_	/	
All‐iron^[^ ^61^ ^]^	H_2_O(0.1M FeSO_4_)	5	0.15
	ethyl acetate/Pyr_14_TFSI(0.1M Fe(acac)_3_)	5	
MV‐TEMPO^[^ ^48^ ^]^	H_2_O/[P_44414_]Cl(TEMPO)	8.77	0.21
	H_2_O/Na_2_SO_4_(MV)	101	
MV/TEMPO^[^ ^49^ ^]^	31.7% PEG, 3.2% Na_2_SO_4_ salt (TEMPO)	8.55	2.64
	1.19% PEG, 17.75% Na_2_SO_4_ salt(MV)	85.7	
Zn/TEMPO^[^ ^50^ ^]^	H_2_O/TEGDME(TEMPO)	6.7	37.5
	H_2_O/MgSO_4_(ZnSO_4_)	/	
V‐VO_2_^[^ ^87^, ^88^ ^]^	V^2+^/V^3+^	≈300	>80
	Nafion 115	≈100	
	VO^2+^/VO_2_^+^	≈400	

#### 3.2.2 Interfacial Ion Migration

Obviously, the interfacial ion migration would significantly affect the performances of biphasic membrane‐less redox batteries, both of ABS and immiscible systems. Based on the previous study,^[^ ^89^, ^90^ ^]^ the interfacial ion migration was influenced by the Galvani potential in the immiscible system and detailed results can be seen in **Table** **5**. For example, for polarized interfaces (water and non‐aqueous solvents are almost immiscible), the priority of ion migration is related to the type of ions. Each ion migration needs to reach a fixed interface potential window value, and the ions within the potential window hardly transfer back and forth between the two phases; For the non‐polarized interface (water and non‐aqueous solvent have some mutual solubility), ion transfer is not controlled by this. In addition, it should be noted that the speed of ion transfer is mainly affected by the nature of the solvent (solvation/desolvation process) and the speed of solvent diffusion.^[^ ^91^ ^]^ For batteries, the electric field formed by the external voltage during the charging and discharging process has a great influence on the ions transfer, while the experiment is only limited to the research on the potential size and direction of the liquid‐liquid interface window at present, and further research is needed on the migration of ions. In addition, as the substitute for IEMs, the liquid‐liquid interface should also have the function of blocking active ions and selectively conducting protons or other exchange salt ions, closely related to the interface electrochemical behavior. Unfortunately, the migration mechanism of interfacial ions has not been systematic investigated, especially for its electrochemical behavior, which has remarkable influence on the battery performance.

**Table 5:** Membrane‐less battery interface potential and the impedance at different current density

Battery‐active species	Solvent	Ion exchange	Interfacial potential/Impedance	Voltage theoretical/actual	Method	Interface potential/voltage
DMFc^[^ ^60^ ^]^	TFT/DCE	Li^+^、ClO_4_^‐^	750mv	0.58/0.8	Four‐electrode	/
	H_2_O					
	TFT/DCE					
pBQ‐H_2_Q^[^ ^55^ ^]^	H_2_O	H^+^	≈300mv	1.4/0.8	Four‐electrode	21%
	Pyr_14_TFSI					
Zn‐Fc^[^ ^64^ ^]^	butyl acetate	Cl^‐^	810 Ω·cm^2^	1.16/0.2	Impedance	/
	H_2_O					
All‐iron^[^ ^61^ ^]^	H_2_O	K^+^、SO_4_^2‐^	153Ω·cm^2^ (d=0.5cm)	1.2/0.75	Impedance	/
	ethyl acetate					
MV‐TEMPO^[^ ^48^ ^]^	H_2_O/[P_44414_]Cl	Na^+^、SO_4_^2‐^	25mv	1.6/1.35	Four‐electrode	15%
	H_2_O					
2,3DMAQ/H_2_Q^[^ ^62^ ^]^	H_2_O	0.1M HCl	≈100mv	1.9/1.35	Four‐electrode	5%
	Pyr_14_TFSI					
OilBlue N/H_2_Q^[^ ^62^ ^]^	H_2_O	0.1M HCl	≈100mv	2.1/1.7	Four‐electrode	4.7%
	Pyr_14_TFSI					
pBQ/H_2_Q^[^ ^62^ ^]^	H_2_O	0.1M HCl 0.1 m TBAPF_6_	≈200mv	1/0.55	Four‐electrode	20%
	PC					
pBQ/TEMPO^[^ ^62^ ^]^	H_2_O	0.1M NaCl	≈0mv	1.5/1.4	Four‐electrode	0
	Pyr_14_TFSI					
pBQ/OH‐TEMPO^[^ ^62^ ^]^	H_2_O	0.5M NaCl	≈40mv	1/0.6	Four‐electrode	4%
	PC					
MV/TEMPO^[^ ^49^ ^]^	H_2_O/PEG	Na_2_SO_4_	>50mv	1.23/0.8	Four‐electrode	4%
	H_2_O					
Zn/TEMPO^[^ ^50^ ^]^	H_2_O/TEGDME	Mg^2+^ Li^+^ Zn^2+^	/	1.5/1	Spectroscopy, quantitative	/
	H_2_O					

**Figure 19:**
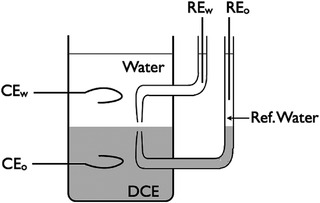
Four‐electrode. Reproduced with permission.^[^ ^92^ ^]^ Copyright 2009, ACS.

Furthermore, experimental technology on the interfacial characteristic of membrane‐less battery was mainly the four‐electrode method (**Figure** **19**) through the measurement of the interfacial potential as well as the impedance between the electrodes. Table 5 lists the value of the interfacial potential (from 25 mv to 750 mv), which occupied larger proportion of most batteries (500≈2000 mv), expected to have a greater impact on the power performance. As for more detailed comparisons, such as the migration ion species, transfer process and transfer rate, there is no detailed study yet.

#### 3.2.3 Mass Transfer

Mass transfer is another key factor affecting the battery performance. It could be speeded up by physical methodto accelerate the redox reactions of active molecules, which could improve the capacity utilization of the battery, and also reduce its cost. It can be found in **Table** **6** that the power of static battery was less than 1 mW cm^−2^, while it was greatly increased in fluidity tests, especially the stirring type with the power performance up to 45.9 mW cm^−2^ and capacity utilization up to 91%. It could be concluded that stirring, may be an effective method to improve the battery performance. The liquid‐liquid interface of membrane‐less batteries, however, is not stable as IEMs, and its disturbance induced by liquid flow would be further exacerbated to break the thermodynamic balance as well as the phase splitting state. Therefore, the application of fluidity strategy is limited due to the characteristic of membrane‐less batteries, and it is also difficult in the structure design of large‐scale battery.

**Table 6:** Comparison of the performances of static, flowing, and stirring batteries

Fluidity	Type	Battery	Concentration (mol L^−1^)	Capacity utilization	Electrode area (cm^2^)	Voltage T/actual	Current (mA cm^−2^)	Power (mW cm^−2^)
static	Immiscible system	All‐iron^[^ ^61^ ^]^	0.05M	/	5	1.2/0.8	0.08	0.06
flow			0.1M	60%	5	1.2/>0.6	0.2	>1.5
static	ABS	Zn/TEMPO^[^ ^50^ ^]^	44mM	25%	1.2	1.5/0.75	51	38.25
stirring				91%	1.2	1.5/0.9	51	45.9

In addition, mass transfer was also restricted by the solvent itself, according to the values of viscosity as listed in Table 7. High viscosity of the solvent, usually as the main factor, is resulting in its relatively low conductivity as well as the difficulty of active material diffusion and reaction kinetics based on the date of diffusion coefficient and kinetic constant in **Table** **7**. It could be inferred that the decrease in the viscosity would improve the battery performances, preliminary tested by mixed ionic liquids and low‐viscosity organic reagents.^[^ ^93^ ^]^ In addition, the reaction kinetics of the active molecules on the electrode surface, the interaction of the active molecules with the solvent, and the factors that support the role of the electrolyte salt still needs more in‐depth study on the influence of these factors. For these influencing factors, Ruiz‐Martín et al.^[^ ^94^ ^]^ conducted an in‐depth study of immiscible systems in the form of computational simulations, which will help deepen the understanding of biphasic batteries.

**Table 7:** The influence of various factors of membrane‐less battery on mass transfer

Battery‐active species	Solvent‐active molecules	Diffusion coefficient D(cm^2^ s^−1^)	kinetic constant K^0^(cm s^−1^)	Viscosity (cp)
pBQ‐H_2_Q^[^ ^55^ ^]^	0.1M HCl (H_2_Q)	4.1 × 10^‐4^	0.379	0.92
	Pyr_14_TFSI (pBQ)	5 × 10^‐5^	1.2 × 10^‐3^	84.33
Zn‐Fc^[^ ^64^ ^]^	30 mol.% Aliquat 336 70 mol.% butyl acetate (0.1M Fc)	8.1 × 10^‐10^ 4.3 × 10^‐10^	1.2 × 10^‐5^	/
	1M ZnCl_2_	10^‐6^	/	
2,3DMAQ/H_2_Q^[^ ^62^ ^]^	0.1M HCl (H_2_Q)	4.1 × 10^‐4^	0.379	/
	Pyr_14_TFSI (2,3DMAQ)	1.4 × 10^‐7^	2.98 × 10^‐4^	/
OilBlue N/H_2_Q^[^ ^62^ ^]^	0.1M HCl (H_2_Q)	4.1 × 10^‐4^	0.379	/
	Pyr_14_TFSI (OilBlueN)	5.10 × 10^‐8^	1.09 × 10^‐4^	2.5
pBQ/H_2_Q^[^ ^62^ ^]^	0.1M HCl (H_2_Q)	4.1 × 10^‐4^	0.379	/
	PC/0.1 m TBAPF_6_ (pBQ)	2.1 × 10^‐6^	3.21 × 10^‐3^	/
pBQ/TEMPO^[^ ^62^ ^]^	0.1M NaCl (TEMPO)	6.6 × 10^‐6^	3.82 × 10^‐4^	/
	Pyr_14_TFSI (pBQ)	5 × 10^‐5^	1.2 × 10^‐3^	/
pBQ/OH‐TEMPO^[^ ^62^ ^]^	0.5M NaCl (OH‐TEMPO)	7.1 × 10^‐6^	5.23 × 10^‐4^	/
	PC/Pyr_14_TFSI (pBQ)	3.9 × 10^‐6^	1.5 × 10^‐3^	/
2,3DMAQ/OH‐TEMPO^[^ ^62^ ^]^	0.5M NaCl (OH‐TEMPO)	7.1 × 10^‐6^	5.23 × 10^‐4^	/
	Pyr_14_TFSI (2,3DMAQ)	1.4 × 10^‐7^	2.98 × 10^‐4^	/
OilBlue N/OH‐TEMPO^[^ ^62^ ^]^	0.5M NaCl (OH‐TEMPO)	7.1 × 10^‐6^	5.23 × 10^‐4^	/
	Pyr_14_TFSI (OilBlue N)	5.10 × 10^‐8^	1.09 × 10^‐4^	/
pBQ/OH‐TEMPO^[^ ^62^ ^]^	0.5M NaCl (OH‐TEMPO)	7.1 × 10^‐6^	5.23 × 10^‐4^	/
	2‐Butanone/0.1 m TBAPF_6_ (pBQ)	/	/	/
pBQ/OH‐TEMPO^[^ ^62^ ^]^	0.5M NaCl (OH‐TEMPO)	7.1 × 10^‐6^	5.23 × 10^‐4^	/
	PC/0.1 m TBAPF_6_ (pBQ)	2.1 × 10^‐6^	3.21 × 10^‐3^	/
MV/TEMPO^[^ ^49^ ^]^	31.7% PEG,3.2% salt (≈0.23 m)	9.09 × 10^‐7^	2.53 × 10^‐3^	/
	1.19% PEG,17.75% salt (≈1.4 m)	5.01 × 10^‐6^	3.41 × 10^‐3^	
Zn/TEMPO^[^ ^50^ ^]^	10% MgSO_4_ TEGDME	/	/	/
	H_2_O			

## 4 Application Prospect

The liquid‐liquid structure has been widely used in industrial extraction, phase transfer catalysis, sensor manufacturing, drug release in pharmacology, and research on simulated biofilms.^[^ ^95^, ^96^, ^97^ ^]^ In recent years, it has gradually been used in the field of energy. the biphasic battery described in the article using the biphasic strategy to eliminate the membrane is a new application, among other things. In addition, the biphasic system still has important applications in other fields.

First, biphasic batteries can solve the problem of hydrogen evolution from the anode of most metal batteries by taking advantage of the non‐aqueous phase, such as lithium‐nickel batteries,^[^ ^98^ ^][^ ^99^ ^][^ ^100^ ^]^ Huiqiao Li^[^ ^98^ ^]^ using 1 m LiClO_4_ in EC/DMC (ethylene carbonate/dimethyl carbonate) as organic electrolyte for metallic lithium anode, and 1 m LiOH + 1 m KOH as aqueous electrolyte for Ni(OH)_2_ cathode. it enabled a wider potential range for the stripping/plating of lithium metal. As a result, the proposed battery exhibited a high voltage of 3.47 V. Similar examples are also found in metal air batteries,^[^ ^101^ ^]^ the non‐polar electrolyte (perfluorooctyltrimethoxysilane, PFTOS) and the polar one (dimethyl sulfoxide, DMSO) were applied to build two‐phase electrolyte interface, which could block the direct contact between Li‐metal electrode and the electrolyte to form a super‐wettability state of PFTOS to Li anode, resulting in the suppression of Li dendrite growth, thereby significantly improving the cycle life of the battery.

Then, “Biphasic strategy” has also been uesd in photo‐ionic cells.^[^ ^102^ ^]^ A photoreaction took place between photo‐excited dye (D) and quencher (Q) redox couples, which were dissolved in the aqueous phase and the organic phase, respectively. And the photoelectron transfer reaction occurred at the liquid‐liquid interface to form a photo‐ionic cell, converting solar energy into electrical energy.^[^ ^103^ ^]^

Finally, in other areas where membrane is realized, biphasic batteries also have considerable application potential such as hydrogen production by electrolysis of water,^[^ ^104^ ^]^ electrodialysis, fuel cell, etc. Although the research on biphasic membrane‐less technology in other aspects used with IEMs has not yet started, it is believed that this strategy would have a good application prospect in fuel cells and so on, especially the biphasic system.

## 5 Conclusion and Outlook

The review summarizes the characteristic of Biphasic membrane‐less redox batteries including of ABS and Immiscible system, and elaborates the principle of phase splitting, as well as the selection rules for solvent and active molecules to facilitate the exploration of the new system.

Based on the design intention of membrane‐less battery, the high‐cost IEMs have been replaced with liquid‐liquid interface and thus is more suitable for large‐scale energy conversion and storage. However, new problems and challenges, such as cross‐contamination and lower power performances, emerged in the membrane‐less battery, which restricted its development. The liquid‐liquid interface has the similar ability of ion selective passing compared with the IEMs, ensuring that active molecules would be confined in the respective phase region without cross‐contamination. For this purpose, various modifications on electrodes, electrolyte, active molecules and supporting electrolytes have been investigated. Although some of them have certain effects, it is still insufficient on the completely inhibition of cross contamination. Fortunately, these modification methods are independent with each other when applied. In the future, several methods can be comprehensively considered on the design of the battery to improve its performance. Another challenge, low power performance has greatly restricted the applications of membrane‐less battery, which was mainly attributed from the solution conductivity and interface ion migration. Especially, the mechanism of interface ion migration has not been systematic studied, which also affected the potential during charge and discharge process. Therefore, in‐depth investigation should be conducted for the proper method to improve its power performance. Unfortunately, there is no reasonable evaluation method to characterize the degree of cross‐contamination and power performance factors of membraneless batteries. We hope to have standardized methods similar to vanadium batteries such as ion permeability and membrane conductivity to further evaluate the future prospects of batteries or clarify the battery mechanism.

Till now, the membrane‐less designs have various defects and cannot be used on a large scale. Among them, the Biphasic membrane‐less redox batteries is a theoretically scalable, low‐cost, and most likely for large‐scale energy storage. Of course, it still faces many problems for application except above. The ionic liquid in water‐ionic liquid battery has high viscosity, low conductivity and high cost, while the use of organic solvents faces volatility, flammability and other issues, so more research is still needed. It is believed that the relevant research would play an important role in the mechanism of membrane‐less batteries, and thus accelerating the development of membrane‐less batteries.

## Conflict of Interest

The authors declare no conflict of interest.
